# Altered expression of the IGF-1 receptor in a tamoxifen-resistant human breast cancer cell line

**DOI:** 10.1038/sj.bjc.6690112

**Published:** 1999-02

**Authors:** J P Parisot, X F Hu, M DeLuise, J R Zalcberg

**Affiliations:** Division of Haematology and Medical Oncology, Peter MacCallum Cancer Institute, Melbourne, Australia; Endocrine and Metabolic Unit, Austin and Repatriation Medical Centre, Melbourne, Australia

**Keywords:** breast cancer, IGF-1R, oestrogen, tamoxifen resistance

## Abstract

The relationship between oestrogen (E_2_) and insulin-like growth factor-one (IGF-1) was examined in both tamoxifen-sensitive (MCF 7/5–21) and tamoxifen-resistant (MCF 7/5–23) subclones of the MCF 7 cell line. Both subclones were grown in defined, serum-free (SF) medium over a period of 7 days with the addition of E_2_ or IGF-1 or a combination of both agents. Growth of both MCF 7/5–21 and 7/5–23 cells was stimulated (245% and 350%, respectively) by E_2_. However, only the growth of MCF 7/5–23 cells was stimulated (266%) by IGF-1. A combination of E_2_ and IGF-1 significantly enhanced MCF 7/5–21 and 7/5–23 cell growth (581% and 695%, respectively). E_2_-induced IGF-1 receptor (IGF-1R) levels (as measured by ^125^ I-IGF-1 binding and Northern analyses) in only MCF 7/5–23 cells. This effect was partially inhibited by tamoxifen. In medium containing serum, the growth of only the MCF 7/5–23 cells was significantly inhibited by the IGF-1R monoclonal antibody, αIR-3. The detection of E_2_-induced expression of IGF-2 using RT-PCR was demonstrated in the MCF 7/5–23 cells. These experiments indicate that E_2_ may sensitize tamoxifen-resistant MCF 7/5–23 cells to the growth stimulatory actions of IGF-2 via up-regulation of the IGF-1R and describes a cell-survival mechanism that may manifest itself as tamoxifen resistance. © 1999 Cancer Research Campaign


British Journal of Cancer (1999) **79**(5/6), 693–700
© 1999 Cancer Research Campaign
Article no. bjoc.1998.0112

### Altered expression of the IGF-1 receptor in a tamoxifen-resistant human breast cancer cell line

**JP Parisot[1], XF Hu[1], M DeLuise[2] and JR Zalcberg[1]**

[1]Division of Haematology and Medical Oncology, Peter MacCallum Cancer Institute, Melbourne, Australia; [2]Endocrine and Metabolic Unit, Austin and Repatriation Medical Centre, Melbourne, Australia

**Summary** The relationship between oestrogen ($E_2$) and insulin-like growth factor-one (IGF-1) was examined in both tamoxifen-sensitive (MCF 7/5–21) and tamoxifen-resistant (MCF 7/5–23) subclones of the MCF 7 cell line. Both subclones were grown in defined, serum-free (SF) medium over a period of 7 days with the addition of $E_2$ or IGF-1 or a combination of both agents. Growth of both MCF 7/5–21 and 7/5–23 cells was stimulated (245% and 350%, respectively) by $E_2$. However, only the growth of MCF 7/5–23 cells was stimulated (266%) by IGF-1. A combination of $E_2$ and IGF-1 significantly enhanced MCF 7/5–21 and 7/5–23 cell growth (581% and 695%, respectively). $E_2$-induced IGF-1 receptor (IGF-1R) levels (as measured by $^{125}$I-IGF-1 binding and Northern analyses) in only MCF 7/5–23 cells. This effect was partially inhibited by tamoxifen. In medium containing serum, the growth of only the MCF 7/5–23 cells was significantly inhibited by the IGF-1R monoclonal antibody, αIR-3. The detection of $E_2$-induced expression of IGF-2 using RT-PCR was demonstrated in the MCF 7/5–23 cells. These experiments indicate that $E_2$ may sensitize tamoxifen-resistant MCF 7/5–23 cells to the growth stimulatory actions of IGF-2 via up-regulation of the IGF-1R and describes a cell-survival mechanism that may manifest itself as tamoxifen resistance.

**Keywords:** breast cancer; IGF-1R; oestrogen; tamoxifen resistance

Oestrogens ($E_2$) stimulate the proliferation of human breast cancer cells predominantly via the oestrogen receptor (ER). However, the clinical effectiveness of antioestrogens such as tamoxifen in ER$^+$ cells is occasionally limited by intrinsic resistance or, more commonly, the acquisition of resistance. The mechanisms underlying the development of anti-oestrogen insensitivity remain elusive, particularly as it is now recognized that tamoxifen resistance is generally not associated with the loss or abnormal function of the ER (Encarnacion and Fuqua, 1994). An alternative mechanism may be the progression of breast tumours to a hormone-independent proliferative state whereby growth factors act as the principal mitogens (Dickson et al, 1993). Of particular interest is the insulin-like growth factor (IGF) family. IGF-1 is a mitogen for human breast cancer cells in vitro (Lippman, 1985), especially in the presence of $E_2$, and acts primarily via a specific cell surface glycoprotein receptor known as the type 1 IGF receptor (IGF-1R; Steele-Perkins et al, 1988).

Evidence for the role of IGF-1 in breast cancer has come from case-control studies that have reported increased circulating levels of IGF-1 in women presenting with breast cancer (Peyrat et al, 1993). Furthermore, the expression of IGF-1R mRNA has been found in most breast cancer cell lines and in over 90% of human breast tumour specimens (Papa et al, 1993). Pekonen et al (1988) have demonstrated a positive correlation between IGF-1R expression and ER and progesterone receptor (PR) content and noted an increase in expression in malignant compared with adjacent normal tissue. Finally, the majority of recent reports have demonstrated that expression of the IGF-1R is vital for the inhibition of apoptosis in tumour cells (Resnicoff et al, 1995; Dunn et al, 1997). It is now widely postulated that IGF-1R signalling is probably more important in the role of tumour cell survival and protection from apoptosis than in mitogenesis (Baserga et al, 1997).

There is also mounting evidence for an interaction between the IGF-1R and ER signalling pathways (Westley and May, 1994). Although IGF-1 can stimulate the proliferation of $E_2$-responsive breast cancer cells on its own, in the presence of $E_2$ a marked synergistic effect is usually observed. This has been associated with the observation that $E_2$ can cause changes in the expression of the IGF-1R and/or its ligands (Stewart et al, 1990; Lee and Yee, 1995). Conversely, it has been shown that IGF-1 can influence the function of the ER. A characteristic response to $E_2$ is the up-regulation of PR expression. However, physiological concentrations of IGF-1 were also found to increase the PR level in MCF 7 breast cancer cells (Katzenellenbogen and Norman, 1990). This stimulation was blocked by an anti-oestrogen suggesting that IGF-1 was acting on a component of the ER pathway. In the absence of any $E_2$, Newton et al (1994) also observed stimulation of ER-mediated reporter-gene activity by IGF-1 in the pituitary tumour cell line, $GH_3$. The mechanism of this ligand-independent activation of the ER is still unclear but it may be involved in the development of tamoxifen resistance.

In this study, we have evaluated the comparative effects of both $E_2$ and IGF-1 on cell growth in an in vitro model of intrinsic tamoxifen-resistant, ER$^+$ breast cancer in defined, serum-free conditions. Our results suggest that the tamoxifen-resistance exhibited by the MCF 7/5–23 cells (relative to the tamoxifen-sensitive MCF 7/5–21 cells) may be associated with IGF-mediated cell proliferation.

*Received 8 December 1997*
*Revised 4 August 1998*
*Accepted 5 August 1998*

*Correspondence to:* JR Zalcberg, Division of Haematology & Medical Oncology, Peter MacCallum Cancer Institute, Locked Bag #1, A'Beckett St, Melbourne 3000, Australia

### MATERIALS AND METHODS

### Reagents

Oestradiol and tamoxifen were purchased from Sigma Chemicals (St Louis, MO, USA). Human recombinant IGF-1 was purchased from GroPep (Adelaide, Australia). Oestradiol and tamoxifen were dissolved in absolute ethanol whereas IGF-1 was dissolved in 10 mM hydrochloric acid (HCl) with 0.1% bovine serum albumin (BSA) (essential fatty acid-free; Sigma Chemicals). $^{125}$I-IGF-1 (S.A: 2800 Ci mmol$^{-1}$) was purchased from NEN-DuPont (Sydney, Australia). Monoclonal antibody to IGF-1R (αIR-3) was obtained from Oncogene Science (Cambridge, MA, USA).

### Cell culture

Two cell lines were used in these studies; a tamoxifen-sensitive (MCF 7/5–21) and a tamoxifen-resistant (MCF 7/5–23) subclone of the parental, sensitive MCF 7 line. The tamoxifen resistance was previously characterized as a 22-fold relative difference (Hu et al, 1993) in the concentration of tamoxifen required to inhibit cell growth by 50% in a growth assay performed in medium containing serum. This characteristic phenotype was also confirmed for the current study. Both subclones express the oestrogen-receptor and were repeatedly found to be *Mycoplasma* free. Stock cultures were maintained in Roswell Park Memorial Institute (RPMI)-1640 (ICN Biomedicals, Aurora, OH, USA) supplemented with 10% fetal bovine serum (FBS: Cytosystems, Sydney, Australia), 6 mM L-glutamine (CSL, Melbourne, Australia), 0.112% $NaHCO_3$ (ICN Biomedicals), 20 mm Hepes (CSL), 10 μg ml$^{-1}$ human insulin (Actrapid HM, Novo Nordisk, Sydney, Australia) and 20 μg ml$^{-1}$ gentamicin (David Bull Laboratories, Melbourne, Australia) at 37°C in a humidified chamber containing 5% carbon dioxide ($CO_2$).

### Cell growth assays: stock (serum-containing) conditions

The relative tamoxifen-resistant growth of the MCF 7/5–23 cells as compared to the tamoxifen-sensitive MCF 7/5–21 cells in stock medium was confirmed using dose–response assays that have been previously described (Hu et al, 1993). Cell growth experiments were also performed in stock medium with the IGF-1R monoclonal antibody (mAb), αIR-3 (Kull et al, 1983). Cells were seeded in 12-well plates (Costar) at a concentration of $1 \times 10^4$ cells/well in 1 ml of stock medium. Forty-eight hours later, fresh stock medium containing αIR-3 (1 μg ml$^{-1}$) or vehicle (1 μg ml$^{-1}$ mouse $IgG_1$) was added and the cells were further incubated for 5 days. The rate of cell proliferation was determined by using the MTT colorimetric cell growth assay kit (Sigma Chemicals) according to the manufacturer's specifications which involved spectrophotometric measurement of cell growth as a function of the mitochondrial activity of living cells.

### Serum-free conditions

A week before each experiment, cells were withdrawn from endogenous steroids present in FBS as originally described by Musgrove and Sutherland (1993). Stock cell cultures were passaged into phenol red-free RPMI-1640 (Gibco-BRL, Grand Island, NY, USA) containing all the supplements described above with the exception of FBS which was substituted with 10% dextran–charcoal-treated FBS (DCC medium; Darbre et al, 1983). The DCC medium was replenished twice over the next 5–7 days by which time cells had reached exponential growth phase and were ready for experimental use.

To assess the effect of $E_2$ or IGF-1, as well as $E_2$ in combination with IGF-1, on growth rates, cells were seeded in 6-well plates (Costar, Cambridge, MA, USA) at an initial concentration of $1 \times 10^5$ cells/well in 2 ml of DCC–FBS medium. Twenty-four hours later, the medium was changed to 5 ml of serum-free, phenol red-free RPMI-1640 supplemented with transferrin (30 nM; Boehringer, Mannheim, Germany), 6 mM L-glutamine, 0.112% $NaHCO_3$, 20 mM Hepes and 20 μg ml$^{-1}$ gentamicin (SF medium) and 10 μg ml$^{-1}$ human insulin. A further 24 h later, SF medium without insulin but containing 1.0 nM of IGF-1, $E_2$ or combinations thereof or equivalent volumes of vehicle solvent were added to the cells as stated in the figure legends. Cell numbers were determined daily after the cells were harvested with a 0.05% trypsin (CSL) and 0.02% EDTA (Flow) solution and viable cells (based on trypan blue dye exclusion) counted using a haemocytometer. All experiments were performed in triplicate and were repeated at least once. Results obtained at the varying concentrations of each mitogen were expressed as a percentage of vehicle control, the mean ± standard error of the mean (SEM).

### IGF-1 binding assays

To determine the role of $E_2$ in up-regulating the IGF-1R, $^{125}$I-IGF-1 binding was measured in monolayers of MCF 7/5–21 and MCF 7/5–23 cells that had been progressively withdrawn from medium containing serum in an identical manner as for the cell growth assays. Prior to the binding assay, the cells were seeded into 25 cm$^2$ culture flasks (Corning Inc., Corning, NY, USA) in DCC medium at a density of $10^6$ cells/flask. After allowing 24 h for cell attachment, the medium was changed to SF medium with insulin. Twenty-four hours later, cells were washed once with sterile phosphate-buffered suline (PBS) and incubated in SF medium containing vehicle, $E_2$, tamoxifen or a combination of $E_2$ and tamoxifen, as stated in the figure legends. Cells were incubated (in quadruplicate) with these reagents for a period of 3–5 days. The monolayers were then washed once with binding assay buffer; sterile PBS containing 0.1% essential fatty acid-free BSA and incubated with 10–15 pM $^{125}$I-IGF-1 in 5 ml of binding assay buffer for up to 16 h at 4°C.

Non-specific binding was determined by incubating cells with $^{125}$I-IGF-1 in the presence of 100 nM unlabelled IGF-1 and 50 μM insulin and was typically 10–20% of total bound radioactivity. Specific binding in the presence of either αIR-3 (10 μg ml$^{-1}$) or insulin (50 μM) was also determined to estimate the proportion of total $^{125}$I-IGF-1 binding to the IGF-1R or to IGF binding proteins (IGFBP). These experiments utilized the distinctive binding properties of the αIR-3 antibody and insulin, both of which will effectively compete for binding with $^{125}$I-IGF-1 to the IGF-1R at high concentrations but not to the IGFBP (Jones and Clemmons, 1995).

After the incubation period, the cells were washed three times with ice-cold binding assay buffer and solubilized with a 0.1% Triton X100, 0.5 M NaOH solution for subsequent determination of bound radioligand using a gamma counter (Cobra-II, Packard, Meriden, CT, USA) with an efficiency of 81% for detecting the $^{125}$I isotope. The statistical significance of the data was established using the analysis of variance method followed by Fisher's multi-comparison test.

© Cancer Research Campaign 1999

### Northern analysis

Total RNA was isolated by acid guanidium thiocyanate/phenol-chloroform extraction (Chomczynski and Sacchi, 1987) of cells that were incubated with test reagents after a week of steroid deprivation as described above. All RNA concentrations were determined spectrophotometrically and the integrity of the RNA was visualized by electrophoretical separation on 1.25% agarose (IBI, New Haven, CT, USA)/2.2 M formaldehyde denaturing gels. RNA was transferred to nylon membranes (MSI, Westboro, MA, USA) by capillary action, UV fixed and stored at –20°C. Human (h)-IGF-1, h-IGF-2 or rat-IGF-1R cDNA probes (generous gifts from Dr Kay Lund) or 18S ribosomal RNA probes (Bresatec, Adelaide, Australia) were labelled using $\alpha$-$^{32}$P-dCTP (NEN-DuPont) by random priming and hybridization was performed in a 50% formamide, 5 × SSPE [0.75 M potassium chloride (NaCl), 50 mM $NaH_2PO_4 \cdot H_2O$, 5 mM EDTA], 0.5% sodium dodecyl sulphate (SDS), 0.35 mg ml$^{-1}$ herring sperm DNA and 5 × Denhardt's solution at 42°C for approximately 16 h. Blots were then washed in a 2 × SSC (0.3 M NaCl, 0.03 M $Na_3$citrate)/0.1% SDS solution for 10 min at 42°C, 1 × SSC/0.1% SDS for 15 min at 42°C and finally in 0.1 × SSC/0.1% SDS for 30 min at 55°C. Blots were exposed to X-ray film (NEN-DuPont) with intensifying screens at –70°C for 3–10 days. Quantification of radiolabelled bands was performed with the Instant Imager system (Packard, Meriden, CT, USA).

### RT-PCR analysis

Poly (A)$^+$ RNA was isolated using the Quickprep Micro mRNA purification kit (Amrad Pharmacia, Sydney, Australia) from cells that were incubated with the reagents indicated in the figure legends under SF conditions. For cDNA synthesis, no more than 100 ng of poly (A)$^+$ RNA was added to 100 ng of random hexamers and double-distilled water (DDW) to a reaction volume of 10 µl. The mixture was incubated at 70°C for 5 min, chilled on ice for 2 min, followed by the addition of 10 µl of the cDNA synthesis master-mix such that the final reactions contained 50 mM Tris-HCl (pH 8.3), 1 mM each of dATP, dGTP, dCTP and dTTP (dNTP), 75 mM KCl, 3 mM magnesium chloride ($MgCl_2$), 5 mM dithiothreitol, 200 units of M-MLV reverse transcriptase (RT) (Gibco-BRL) and 10 units of RNase inhibitor (Gibco-BRL). The reaction mix was incubated at 37°C for 60 min, diluted with 30 µl of DDW and terminated by a final 5 min incubation at 90°C.

Both IGF-2 and histone 3.3 (H3.3) mRNA expression were assayed by semi-quantitative reverse transcriptase polymerase chain reaction (RT-PCR) using specific primers for amplification. The IGF-2 primers were sense GGCTTCTACTTCAGCAGGC (exon 6) and antisense GTGGGTAGAGCAATCAGGG (exon 8) yielding a 344 bp product. The H3.3 primers were sense CCACTGAACTTCTGATTCGC (exon 2) and antisense GCGTGCTAGCTGGATGTCTT (exon 3) yielding a 214 bp product. The ubitiquously expressed H3.3 gene was used as an endogenous internal control for IGF-2 in the RT-PCR assay to check RNA integrity and loading. For IGF-2 detection, one-tenth of the reaction volume obtained after cDNA synthesis (5 µl) was diluted with a PCR-mixture (up to a final volume of 50 µl) such that the final reaction consisted of 0.5 µM each of the primer pair, 20 mM Tris-HCl (pH 8.3), 50 mM KCl, 0.1 mg ml$^{-1}$ BSA, 1.5 mM $MgCl_2$, 0.2 mM of each dNTP and 1.0 unit of *Taq* polymerase (Promega, Sydney, Australia). The PCR mixture was overlaid with 50 µl of mineral oil (Sigma) to prevent evaporation and then underwent a thermal cycling protocol consisting of 35 cycles of template denaturation at 95°C for 1 min, primer-template annealing at 57°C for 1 min and polymerase extension at 72°C for 1 min, followed by a final extension at 72°C for 10 min on an Omnigene thermal cycler (Hybaid, Melbourne, Australia).

The H3.3 gene was always assayed simultaneously using the identical protocol as described above for the IGF-2 gene with the only exception being that the cDNA template was diluted 1:10 before PCR cycling due to the relatively high abundance of H3.3 expression compared to IGF-2. The PCR cycling parameters were determined from preliminary experiments where we demonstrated that amplification of both IGF-2 and H3.3 levels was in the exponential phase of PCR at 35 cycles for the concentrations of template that were used. Thus, levels of IGF-2 expression detected by RT-PCR were normalized for levels of H3.3 for every sample and comparisons of the IGF-2/H3.3 ratios between samples were made only within the same assay. The negative cDNA template control (DDW) was subjected to 40 cycles of PCR with no amplification of H3.3 or IGF-2 detected. Quantification of IGF-2 and H3.3 levels were performed using Molecular Analyst software on a UV-Gel-Documentation instrument (BioRad, Sydney, Australia) analysing the PCR product signal after size-fractionation on a 1.5% agarose gel.

### RESULTS

### Tamoxifen-sensitive and resistant breast cancer cells are $E_2$-responsive but differ in IGF-1 sensitivity

In the presence of tamoxifen, the proliferation of both MCF 7 subclones was significantly altered over a time-course of 7 days. In dose–response growth assays, the concentrations of tamoxifen that resulted in a 50% inhibition of cell growth ($IC_{50}$) were 66.1 nM and 1.08 µM in the MCF 7/5–21 and 7/5–23 subclones (Figure 1), respectively. This represented a 17-fold difference in sensitivity to tamoxifen, results that were similar to our earlier studies (Hu et al, 1993) which also demonstrated that the ER number and affinity of binding to $E_2$ was not different between the two cell lines (Parisot et al, 1995).

Both subclones were successfully cultured in defined, serum-free conditions after approximately a week of serum withdrawal. Under these conditions, the response to the mitogens $E_2$ and IGF-1 could be determined with negligible interference from residual $E_2$ and/or growth factors normally present in serum. By day 7 of cell culture, the growth of the tamoxifen-sensitive, MCF 7/5–21 cells was stimulated 2.5-fold by $E_2$ (compared to vehicle controls) at a physiological concentration of 1 nM (Table 1). The ethanol vehicle had a minimal effect on cell growth (data not shown). By itself, IGF-1 (1 nM) had no effect on the growth of these cells compared to vehicle controls; however, the combination of $E_2$ and IGF-1 markedly enhanced cell growth almost sixfold.

The growth of MCF 7/5–23 cells was stimulated 3.5-fold by $E_2$ by day 7 of the experiment. When $E_2$ and IGF-1 were combined, enhanced proliferation of MCF 7/5–23 cells was also demonstrated (almost sevenfold) compared with either agent alone (Table 1). In contrast to MCF 7/5–21 cells, IGF-1 alone enhanced the growth of MCF 7/5–23 cells to levels similar to those attained with $E_2$. A more detailed investigation of the response of both MCF 7/5–21 and MCF 7/5–23 cells to different doses of IGF-1 (up to 100 nM) over a 7-day period demonstrated that only the proliferation of the tamoxifen-resistant, MCF 7/5–23 cells increased in a

© Cancer Research Campaign 1999

**Table 1** Effect of $E_2$, IGF-1 or a combination of both agents on MCF 7/5–21 and MCF 7/5–23 cell proliferation

| Cell line | $E_2$ | IGF-1 | $E_2$ + IGF-1 |
|---|---|---|---|
| MCF 7/5–21[a] | 245 ± 27[b] | 119 ± 7 | 581 ± 26[b] |
| MCF 7/5–23[a] | 350 ± 51[b] | 266 ± 19[b] | 695 ± 82[b] |

Cells were plated in 6-well plates after being withdrawn from oestrogens as described in Materials and Methods. Triplicate wells were then treated for 7 days with SF medium supplemented with either vehicle (0.1% ethanol), $E_2$ ($10^{-9}$ M), IGF-1 ($10^{-9}$ M) or a combination of $E_2$ + IGF-1 ($10^{-9}$ M). Cell numbers were measured and results were expressed as a % of the number of viable cells counted in the absence of any drug (vehicle only), with each number representing the mean ± SEM of three separate experiments. [a]Expressed as a % of vehicle control. [b]$P < 0.05$ by ANOVA and Fisher's multi-comparison statistical significance tests.

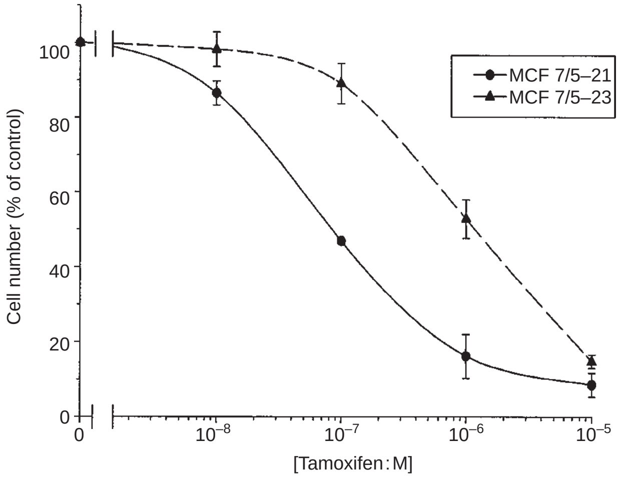

**Figure 1** The effect of tamoxifen on MCF 7/5–21 and 7/5–23 cell proliferation. Cells from both subclones were seeded in stock medium and allowed to reach the exponential growth phase (48 h). The subclones were incubated with stock medium supplemented with either vehicle (0.1% ethanol) or a range of tamoxifen concentrations ($10^{-8}$–$10^{-5}$ M) for a further 5 days as described in Materials and methods. Results are expressed as a % of the number of viable cells counted in the absence of any drug (vehicle only) with each bar representing the mean ± SEM of three separate experiments, each performed in triplicate dose-dependent fashion (data not shown). Furthermore, tamoxifen had only a slight inhibitory effect on the IGF-1-mediated growth of MCF 7/5–23 cells (data not shown).

### $E_2$ up-regulates IGF-1 binding in tamoxifen-resistant but not in tamoxifen-sensitive breast cancer cells

To determine whether the enhanced stimulatory effect of the combination of $E_2$ and IGF-1 on the growth of both MCF 7/5–23 cells and MCF 7/5–21 cells could be attributed to an alteration in the expression of the IGF-1R by $E_2$, we measured the effects of $E_2$, tamoxifen alone and $E_2$ plus tamoxifen on $^{125}$I-IGF-1 binding. A twofold increase in the specific binding of $^{125}$I-IGF-1 was demonstrated in the MCF 7/5–23 cell line after exposure to 1 nM $E_2$ (Figure 2A). A similar effect was not observed in the MCF 7/5–21 cell line (Figure 2A). Whereas, tamoxifen alone had no effect on the level of $^{125}$I-IGF-1 binding in either cell line, this antioestrogen was able to partially inhibit the effect of 1 nM $E_2$ on the binding of $^{125}$I-IGF-1 in the MCF 7/5–23 cell line (Figure 2A), suggesting that the increase in binding was mediated via the ER. Specific $^{125}$I-IGF-1 binding measured in the presence of either αIR-3 or insulin, both of which do not bind to IGFBP, demonstrated that after treatment with $E_2$ the increase in $^{125}$I-IGF-1 in MCF 7/5–23 cells was predominantly to the IGF-1R (Figure 2B). Again, this effect of $E_2$ on $^{125}$I-IGF-1 binding to the IGF-1R was not observed with the MCF 7/5–21 cell line (data not shown).

### Northern blot analysis detects the up-regulation of IGF-1R gene expression by $E_2$ in tamoxifen-resistant breast cancer cells

To examine whether the effects of $E_2$ on $^{125}$I-IGF-1 binding correlated with an effect on the expression of IGF-1R mRNA, we carried out Northern blot analyses of total RNA isolated from MCF 7/5–21 and 7/5–23 cells as described in the Materials and methods. The hybridization of the IGF-1R probe to both the 11 and 7 kilobase mRNA transcripts described in human tissues (Ullrich et al, 1986) is evident in MCF 7/5–23 cells as illustrated in Figure 3. The rat IGF-1R cDNA probe was used to detect hIGF-IR mRNA in these cell lines as it shares 94% sequence homology to its human counterpart (Werner et al, 1989). In the tamoxifen-resistant MCF 7/5–23 cells, 4–24 h of exposure to 1 nM $E_2$ in SF medium resulted in a rapid induction of IGF-1R mRNA expression (Figure 3). This effect was still evident when expression of IGF-1R mRNA was measured after 120 h of $E_2$ exposure (Figure 3) but was not as marked as in the shorter time course. No effect of $E_2$ in the tamoxifen-sensitive MCF 7/5–21 cells was observed at any time period (data not shown). When the MCF 7/5–23 cells were incubated with $E_2$ and tamoxifen simultaneously for 120 h, the induction of the IGF-1R mRNA was moderately inhibited (Figure 3), a result which correlated with data from the IGF-1 binding assays over the same time-course. The effect of tamoxifen alone on IGF-1R mRNA levels in MCF 7/5–23 cells was not significantly different from vehicle controls overall (three experiments).

### RT-PCR analysis detects IGF-2 mRNA induced by $E_2$ in both breast cancer cell lines but no IGF-1 gene expression

Northern data analysis revealed no IGF-1 or IGF-2 mRNA in either cell line grown in SF conditions (data not shown). However, the highly sensitive RT-PCR technique was able to detect IGF-2 expression in both the MCF 7/5–21 cells and the MCF 7/5–23 cells when both cell lines were incubated with vehicle (0.1% ethanol) in SF medium (Figure 4). Relative to the corresponding H3.3 levels for each sample, the basal expression of IGF-2 was increased by 5 days of $E_2$ exposure in both the MCF 7/5–21 cells (161 ± 4%) and the MCF 7/5–23 cells (240 ± 42%). Co-incubation of both cell lines with $E_2$ and tamoxifen reduced the expression of IGF-2 to near basal levels. Under no condition did we detect the expression of IGF-1 mRNA in either cell line using RT-PCR (data not shown).

### αIR-3 inhibits the growth of only tamoxifen-resistant breast cancer cells

Experiments measuring the effect of the IGF-1R mAb, αIR-3, on the rate of cell proliferation in stock medium demonstrated that whereas the antibody had no significant effect on MCF 7/5–21 cell

 © Cancer Research Campaign 1999

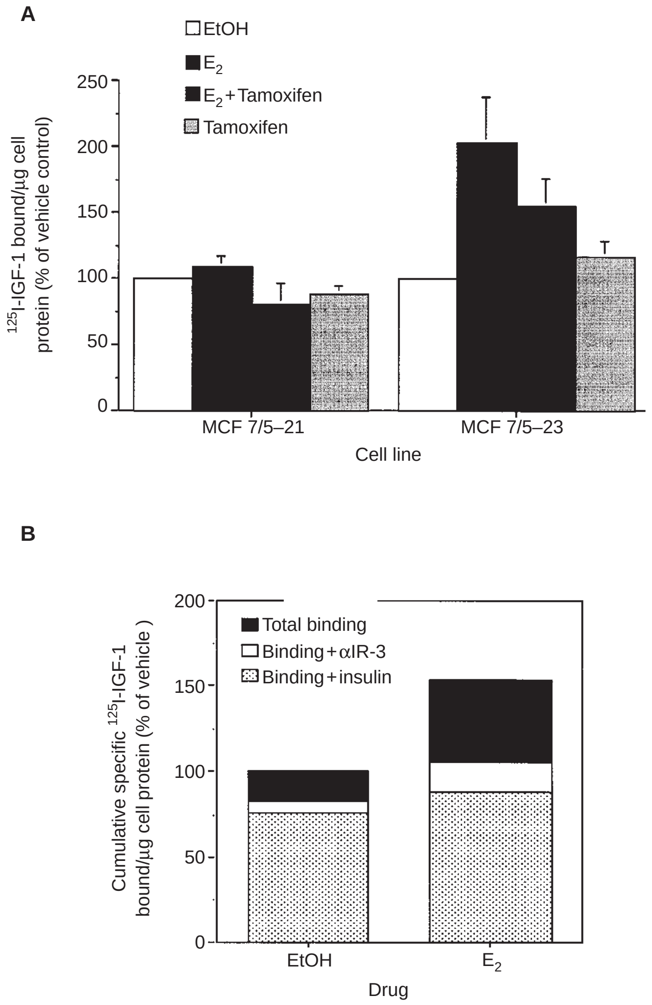

**Figure 2** The effect of $E_2$ and tamoxifen on IGF-1 binding to breast cancer cells in serum-free conditions. After the cells were withdrawn from oestrogens as described in Materials and methods, binding of $^{125}$I-IGF-1 was measured after 5 days incubation in SF medium with the following additions: vehicle (0.1% ethanol), $E_2$ ($10^{-9}$ M), $E_2$ ($10^{-9}$ M) + tamoxifen ($10^{-6}$ M) and tamoxifen ($10^{-6}$ M). Near-confluent monolayers of MCF 7/5–21 and 7/5–23 cells were incubated for 16 h at 4°C with 10–20 pM of $^{125}$I-IGF-1 ± 100 nM IGF-1 and 50 μM insulin to determine specific, total binding (**A**) or plus αIR-3 (10 μg ml$^{-1}$) or insulin (50 μM) to determine specific binding to the IGF-1R or IGFBP components in the MCF 7/5–23 cell line (**B**). The cells were washed, lysed and radioactivity counted. Results are expressed as a % of the vehicle control values and are normalized for cell protein content. Each bar represents the mean ± SEM of six separate experiments with a significant ($P < 0.05$) difference in IGF-1 binding for the group $E_2$ vs EtOH in MCF 7/5–23 cells only

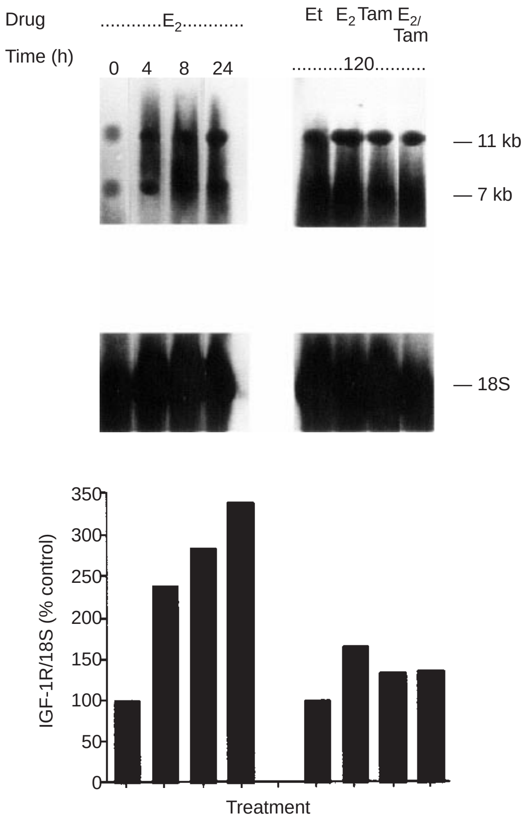

**Figure 3** Northern blot analyses of IGF-1R mRNA expression in MCF 7/5–23 cells. Total RNA was extracted from cells withdrawn from oestrogens and treated in SF medium for 0–120 h with the following additions as indicated: vehicle (0.1% ethanol), $E_2$ ($10^{-9}$ M), tamoxifen ($10^{-6}$ M) and $E_2$ ($10^{-9}$ M) + tamoxifen ($10^{-6}$ M). At least 30 μg of total RNA was size-fractionated by gel electrophoresis, transferred to a nylon filter, hybridized with the α- $^{32}$P-dCTP-rIGF-1R cDNA probe, autoradiographed for up to 10 days and quantified by image analysis. Results from the representative experiment are expressed as a % of the vehicle control and have been corrected for loading by normalizing for 18S ribosomal RNA levels. Similar results were observed in repeated experiments growth (Figure 5), significant growth inhibitory effects were demonstrated in the MCF 7/5–23 cells. The MTT assay was employed in these experiments as a measurement of the antibody's effect on actively dividing cells as opposed to counting the number of viable cells.

### DISCUSSION

We have previously demonstrated that compared to its sister subclone MCF 7/5–21, the relative tamoxifen resistance of the MCF 7/5–23 cells was not explained by the loss of expression of the ER or by a lower affinity of the ER for tamoxifen (Parisot et al, 1995). Thus we reasoned that the tamoxifen-resistant phenotype may have been due to the $E_2$-independent, autonomous growth of MCF 7/5–23 cells. In order to test this hypothesis and examine the role of mitogens such as $E_2$ and IGF-1, we measured the growth of cells in defined, serum-free conditions to avoid potential contamination by residual $E_2$ or other growth factors present in charcoal-stripped FBS. In growth assays completed over a period of 7 days, we were able to demonstrate a proliferative response to $E_2$ (in the absence of insulin) in both the MCF 7/5–21 and MCF 7/5–23 subclones. In this study, IGF-1 alone was not mitogenic in the MCF 7/5–21 cells but was significantly mitogenic in the MCF 7/5–23 cells (Table 1).

With the exception of earlier studies by Huff et al (1986) and Karey and Sirbasku (1988), more recent data have shown that IGF-1 alone has only a slight effect on MCF 7 cell proliferation. The larger effects of IGF-1 in these earlier studies were most likely due to the low levels of residual $E_2$ in the culture medium or the presence of the phenol-red dye, a weak $E_2$ (Berthois et al, 1986). Hence, studies which used a more severe $E_2$-withdrawal regimen lasting almost a week, as well as phenol-red free medium, observed very small effects of IGF-1 on MCF 7 cell proliferation (Stewart et al, 1990, 1992; Thorsen et al, 1992; Wiseman et al, 1993). However, all of these studies were able to demonstrate a marked synergistic effect of $E_2$ and IGF-1 on MCF 7 cell proliferation despite the

© Cancer Research Campaign 1999

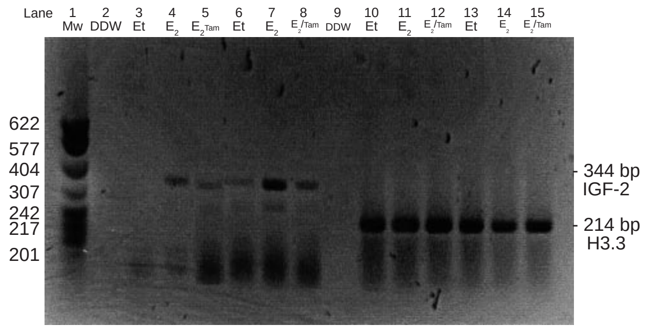

**Figure 4** RT-PCR analysis of IGF-II and H3.3 mRNA expression. Poly (A)+ RNA was reverse-transcribed and one-tenth of the reaction volume was amplified with specific primers for IGF-2 and histone 3.3 for 35 cycles. Following PCR, the amplified products were electrophoresed in 1.5% agarose gels stained with ethidium bromide (0.5 mg ml) and the fluorescence quantified with image analysis software. Legend: pBR322/*Msp*1 Mw ladder (lane 1); DDW (lane 2, 9), MCF 7/5–21 cells and MCF 7/5–23 cells incubated with vehicle (0.1% ethanol), $E_2$ ($10^{-9}$ M) and $E_2$ ($10^{-9}$ M) + tamoxifen ($10^{-6}$ M), respectively, with amplification for IGF-2 (5–21: lanes 3–5; 5–23: lanes 6–8) and histone 3.3 (5–21: lanes 10–12; 5–23: lanes 13–15). Data from this representative experiment was similar to those observed in repeated experiments.

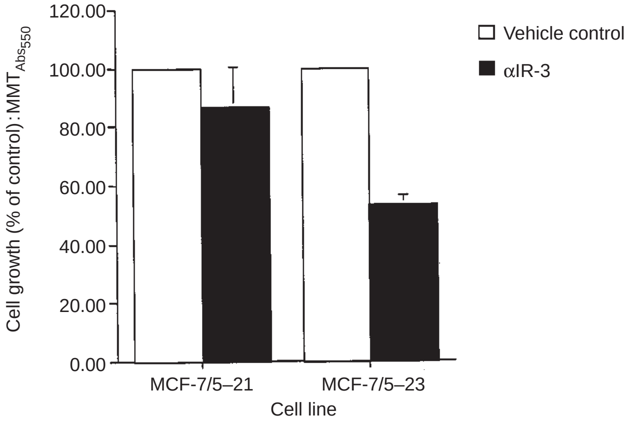

**Figure 5** Inhibition of cell growth by the IGF-1R monoclonal antibody (αIR-3). MCF 7/5–21 and 7/5–23 cells were plated and grown for 48 h in RPMI-1640 with 10% FBS before the addition of αIR-3 (1 μg ml–1). Five days later, the rate of cell proliferation was determined by spectrophotometric measurement of MTT dye reduction. Results are expressed as a % of the absorbance measured in the presence of vehicle only (1 μg ml–1 mouse $IgG_1$) with each bar representing the mean ± SEM of two separate experiments with a significant ($P < 0.05$) difference for MCF 7/5–23 cells only.

insignificant effect of IGF-1 alone, as was observed in this study with the MCF 7/5–21 cells.

This synergy between $E_2$ and IGF-1 on ER+ breast cancer cell proliferation has been previously attributed to the up-regulation of the IGF-1R by $E_2$ (Stewart et al, 1990; Westley and May, 1994; Huynh et al, 1996; Clarke et al, 1997). Similarly, our results demonstrated that the addition of $E_2$ doubled $^{125}$I-IGF-1 binding in the tamoxifen-resistant MCF 7/5–23 cells over a 5-day period. That the $E_2$-induced increase in $^{125}$I-IGF-1 binding in MCF 7/5–23 cells was due to the induction of IGF-1R mRNA by $E_2$ was confirmed using Northern blot analyses. It was found that $E_2$ exposure for 24 h increased the expression of IGF-1R mRNA about threefold in MCF 7/5–23 cells and was still relatively elevated 4 days later, data which correlated with the results of the binding assay. Yet, peculiarly, we were unable to demonstrate this effect in the tamoxifen-sensitive MCF 7/5–21 cells.

The role of the IGFBP cannot be discounted as it may mask changes in the level of the IGF-1R in the MCF 7/5–21 cells when measured by $^{125}$I-IGF-1 binding assays. However, preliminary examination of specific $^{125}$I-IGF-1 binding to the IGF-1R by using the αIR-3 antibody or a high concentration of insulin revealed that there was no effect of $E_2$ in the MCF 7/5–21 cells but $^{125}$I-IGF-1 binding to the IGF-1R was still increased in the MCF 7/5–23 cells. Hence, the induction of $^{125}$I-IGF-1 binding was most probably indicative of an increment in IGF-1R number and not a measurement of membrane-associated IGFBP activity. Nevertheless, we did observe that up to 80% of specific $^{125}$I-IGF-1 binding in these MCF 7 subclones was to the IGFBP as has been previously reported (Kleinman et al, 1995).

The data also demonstrate that IGF-1R signalling is activated during the culture of either of the subclones in serum-containing medium because of the ability of the IGF-1R mAb, αIR-3, to inhibit the growth of the cells. Although the inhibition of MCF 7/5–21 cell growth by αIR-3 was not significant, the mean level of cell growth inhibition was 20%, a range comparable to that observed with the MCF 7 cells at a concentration of 1 μg ml–1 antibody (Rohlik et al, 1987; Arteaga and Osborne, 1989). In contrast, αIR-3 (1 μg ml–1) significantly inhibited the growth of the MCF 7/5–23 cells by 50%, implicating a higher level of IGF-1R activity in this cell line.

Although others have reported over-expression of the IGF-1R in breast cancer (Pekonen et al, 1988; Papa et al, 1993) there are only limited data regarding the association of this finding with the development of tamoxifen resistance in ER+ breast cancer (Wiseman et al, 1993). Decupis et al (1995) observed that, although $E_2$ increased the number of IGF-1R in tamoxifen-resistant MCF 7/LCC2 cells, it did not do likewise in tamoxifen-resistant MCF 7/LY-2 cells. Similarly, van den Berg et al (1996) observed that $E_2$ failed to up-regulate the IGF-1R in the tamoxifen-resistant ZR-75-9al cells, which expressed a lower level of the IGF-1R (McCotter et al, 1996). This difference between the tamoxifen-resistant cell lines is presumably related to the level of expression of the ER and the degree of $E_2$ responsiveness.

Wiseman et al (1993) suggested that the development of tamoxifen resistance occurred via the agonistic (oestrogenic) properties of tamoxifen increasing the expression of the IGF-1R. They reported an increase in $^{125}$I-IGF-1 binding by tamoxifen in a tamoxifen-resistant subclone of MCF 7 cells. In contrast, I detected no agonistic effects of tamoxifen in the MCF 7/5–23 cells. However, our data indicated that tamoxifen was unable to completely inhibit $E_2$-induced $^{125}$I-IGF-1 binding to basal levels. When tamoxifen was co-incubated with $E_2$, the resultant $^{125}$I-IGF-1 binding was partially inhibited compared with $E_2$ alone (Figure 2A) and tamoxifen partially inhibited $E_2$-induced IGF-1R mRNA expression (Figure 3), suggesting that tamoxifen was predominantly acting as an $E_2$ antagonist in these cells and under these conditions but was not fully effective.

A possible explanation for the difference in these observations may be the methods used to generate these tamoxifen-resistant cell lines. Although the results of Wiseman et al (1993) were obtained with subclones selected in the continued presence of tamoxifen, the tamoxifen-resistant MCF 7/5–23 cell line reported herein represented a spontaneously generated subclone obtained by maintaining the genetically unstable MCF 7 cells in continuous exponential growth (Reddel et al, 1988). No other selective pressure was used to achieve tamoxifen resistance (defined relative to its sister subclone MCF 7/5–21, generated concurrently).

Many investigators have shown an increase in $^{125}$I-IGF-1 binding or IGF-1R mRNA after $E_2$ treatment in the MCF 7 cell

 *© Cancer Research Campaign 1999*

line (Freiss et al, 1990; Stewart et al, 1990; Decupis et al, 1995; Huynh et al, 1996; Clarke et al, 1997). It is unclear to us why $E_2$ failed to increase $^{125}$I-IGF-1 binding or IGF-1R mRNA levels in the MCF 7/5–21 cells, given that their rate of proliferation was also increased synergistically by co-incubation with $E_2$ and IGF-1. Another possible explanation could be that the synergy between $E_2$ and IGF-1 is also dependent on other mechanisms besides the up-regulation of the IGF-1R by $E_2$. Recently, it has been proposed that the synergy between $E_2$ and IGF-1 may be mediated by increased activation of the ER through IGF-1R cross-talk. Stimulation of the IGF-1R has been implicated in the increase of ER-phosphorylation through the Ras-MAPK cascade of the IGF-1R signalling pathway (Kato et al, 1995) resulting in enhanced ER-mediated cell proliferation.

The detection of IGF-1R mRNA expression in the MCF 7/5–23 cells and their sensitivity to IGF-1 lead us to question whether the IGF-mediated proliferation of these cells was via an autocrine mechanism. However, we could detect no IGF-1 mRNA expression using firstly Northern blot analysis or the more sensitive RT-PCR method. In fact, IGF-1 mRNA has not been detected using RT-PCR in a large number of breast cancer cell lines (Quinn et al, 1996), although IGF-2 mRNA has been detected both in T47D cells and late passage MCF 7 cells (Yee et al, 1988). Using RT-PCR, we demonstrated that both of our subclones expressed IGF-2 mRNA and we were able to identify a significant, $E_2$-induced increase in IGF-2 expression primarily in the MCF 7/5–23 cells (Figure 4). Thus, IGF-2, a ligand that has a moderately high affinity for the IGF-1R, may act as an autocrine regulator of cell growth in these cell lines but we have not as yet demonstrated the presence of immunoreactive IGF-2. This may be especially important for the MCF 7/5–23 cells, which we have shown are responsive to IGF-1 and express IGF-1R that is up-regulated by physiological concentrations of $E_2$. Interestingly, immunoreactive IGF-2 activity has been detected in MCF 7 (Lee et al, 1994*a*) and T47D cells (Osborne et al, 1989) and in a tamoxifen-resistant MCF 7 subclone (Lee et al, 1994*b*) developed by Toi et al (1993).

Finally, in MCF 7/5–23 cells the up-regulation of IGF-1R expression by $E_2$ only partially inhibited by tamoxifen combined with the sensitivity of these cells to IGF-1, may be the explanation for the relative tamoxifen-resistance of MCF 7/5–23 cells compared to MCF 7/5–21 cells which are unable to bypass growth inhibition by tamoxifen through the IGF-1R pathway.

### REFERENCES

Arteaga CL and Osborne CK (1989) Growth inhibition of human breast cancer cells in vitro with an antibody against the type I somatomedin receptor. *Cancer Res* **49**: 6237–6241

Baserga R, Hongo A, Rubini M, Prisco M and Valentinis B (1997) The IGF-1 receptor in cell growth, transformation and apoptosis. *Biochim Biophys Acta* **1332**: F105–F126

Berthois Y, Katzenellenbogen J and Katzenellenbogen B (1986) Phenol red in tissue culture media is a weak estrogen: implications concerning the study of estrogen-responsive cells in culture. *Proc Natl Acad Sci USA* **83**: 2496–2500

Chomczynski P and Sacchi N (1987) Single-step method of RNA isolation by acid guanidinium thiocyanate-phenol-chloroform extraction. *Anal Biochem* **162**: 156–159

Clarke R, Howell A and Anderson E (1997) Type I insulin-like growth factor receptor gene expression in normal human breast tissue treated with oestrogen and progesterone. *Br J Cancer* **75**: 251–257

Darbre P, Yates J, Curtis S and King R (1983) Effect of estradiol on human breast cancer cells in culture. *Cancer Res* **43**: 349–354

Decupis A, Noonan D, Pirani P, Ferrera A, Clerico L and Favoni RE (1995) Comparison between novel steroid-like and conventional nonsteroidal antioestrogens in inhibiting oestradiol- and IGF-1-induced proliferation of human breast cancer-derived cells. *Br J Pharmacol* **116**: 2391–2400

Dickson RB, Johnson MD, el Ashry D, Shi YE, Bano M, Zugmaier G, Ziff B, Lippman ME and Chrysogelos S (1993) Breast cancer: influence of endocrine hormones, growth factors and genetic alterations. *Adv Exp Med Biol* **330**: 119–141

Dunn S, Hardman R, Kari F and Barrett J (1997) Insulin-like growth factor 1 (IGF-1) alters drug sensitivity of HBL100 human breast cancer cells by inhibition of apoptosis induced by diverse anticancer drugs. *Cancer Res* **57**: 2687–2693

Encarnacion CA and Fuqua SA (1994) Oestrogen receptor variants in breast cancer. *Cancer Treat Res* **71**: 97–109

Freiss G, Rochefort H and Vignon F (1990) Mechanisms of 4-hydroxytamoxifen anti-growth factor activity in breast cancer cells: alterations of growth factor receptor binding sites and tyrosine kinase activity. *Biochem Biophys Res Commun* **173**: 919–926

Hu XF, Veroni M, De Luise M, Wakeling A, Sutherland R, Watts CK and Zalcberg JR (1993) Circumvention of tamoxifen resistance by the pure antioestrogen ICI 182,780. *Int J Cancer* **55**: 873–876

Huff K, Kaufman D, Gabbay KH, Spencer EM, Lippman ME and Dickson RB (1986) Secretion of an insulin-like growth factor-I-related protein by human breast cancer cells. *Cancer Res* **46**: 4613–4619

Huynh H, Nickerson T, Pollak M and Yang XF (1996) Regulation of insulin-like growth factor I receptor expression by the pure antioestrogen ICI 182,780. *Clin Cancer Res* **2**: 2037–2042

Jones JI and Clemmons DR (1995) Insulin-like growth factors and their binding proteins: biological actions. *Endocr Rev* **16**: 3–34

Karey K and Sirbasku D (1988) Differential responsiveness of human breast cancer cell lines MCF-7 and T47D to growth factors and 17 beta-estradiol. *Cancer Res* **48**: 4083–4092

Kato S, Endoh H, Masuhiro Y, Kitamoto T, Uchiyama S, Sasaki H, Masushige S, Gotoh Y, Nishida E, Kawashima H, Metzger D and Chambon P (1995) Activation of the oestrogen receptor through phosphorylation by mitogen-activated protein kinase. *Science* **270**: 1491–1494

Katzenellenbogen BS and Norman MJ (1990) Multihormonal regulation of the progesterone receptor in MCF-7 human breast cancer cells: interrelationships among insulin/insulin-like growth factor-I, serum, and oestrogen (published erratum appears in *Endocrinology* 1990; **126**: 3217.) *Endocrinology* **126**: 891–898

Kleinman D, Karas M, Roberts CJ, LeRoith D, Phillip M, Segev Y, Levy J and Sharoni Y (1995) Modulation of insulin-like growth factor I (IGF-1) receptors and membrane-associated IGF-binding proteins in endometrial cancer cells by estradiol. *Endocrinology* **136**: 2531–2537

Kull-Jr FC, Jacobs S, Su Y, Svoboda M, Van Wyk J and Cuatrecasas P (1983) Monoclonal antibodies to receptors for insulin and somatomedin-C. *J Biol Chem* **258**: 6561–6566

Lee AV and Yee D (1995) Insulin-like growth factors and breast cancer. *Biomed Pharmacother* **49**: 415–421

Lee AV, Darbre P and King R (1994*a*) Processing of insulin-like growth factor-II (IGF-II) by human breast cancer cells. *Mol Cell Endocrinol* **99**: 211–220

Lee AV, Toi M and Yee D (1994*b*) Tamoxifen resistant MCF 7 cells exhibit increased expression of insulin-like growth factor-II (IGF-II). *Breast Cancer Res Treat* **32S**: 57

Lippman ME (1985) Growth regulation of human breast cancer. *Clin Res* **47**: 375–382

McCotter D, Vandenberg HW, Boylan M and McKibben B (1996) Changes in insulin-like growth factor-I receptor expression and binding protein secretion associated with tamoxifen resistance and oestrogen independence in human breast cancer cells in vitro. *Cancer Lett* **99**: 239–245

Musgrove EA and Sutherland RL (1993) Acute effects of growth factors on T47D breast cancer cell cycle progression. *Eur J Cancer* **29A**: 2273–2279

Newton CJ, Trapp T, Pagotto U, Renner U, Buric R and Stalla GK (1994) The oestrogen receptor modulates growth of pituitary tumour cells in the absence of exogenous oestrogen. *J Mol Endocrinol* **12**: 303–312

Osborne C, Coronado EB, Kitten LJ, Arteaga CL, Fuqua SA, Ramasharma K, Marshall M and Li CH (1989) Insulin-like growth factor-II (IGF-II): a potential autocrine/paracrine growth factor for human breast cancer acting via the IGF-1 receptor. *Mol Endocrinol* **3**: 1701–1709

Papa V, Gliozzo B, Clark GM, McGuire WL, Moore D, Fujitayamaguchi Y, Vigneri R, Goldfine ID and Pezzino V (1993) Insulin-like growth factor-I receptors are overexpressed and predict a low risk in human breast cancer. *Cancer Res* **53**: 3736–3740

Parisot JP, Hu XF, Sutherland R, Wakeling A, Zalcberg J and DeLuise M (1995) The pure antioestrogen ICI 182,780 binds to a high-affinity site distinct from the oestrogen receptor. *Int J Cancer* **62**: 480–484

© Cancer Research Campaign 1999 

Pekonen F, Partanen S, Makinen T and Rutanen E (1988) Receptors for epidermal growth factor and insulin-like growth factor I and their relation to steroid receptors in human breast cancer. *Cancer Res* **48**: 1343–1347

Peyrat J, Bonneterre J, Hecquet B, Vennin P, Louchez M, Fournier C, Lefebvre J and Demaille A (1993) Plasma insulin-like growth factor-1 (IGF-1) concentrations in human breast cancer. *Eur J Cancer* **29A**: 492–497

Quinn KA, Treston AM, Unsworth EJ, Miller MJ, Vos M, Grimley C, Battey J, Mulshine JL and Cuttitta F (1996) Insulin-like growth factor expression in human cancer cell lines. *J Biol Chem* **271**: 11477–11483

Reddel RR, Alexander IE, Masfumi K, Shine J and Sutherland R (1988) Genetic instability and the development of steroid hormone insensitivity in cultured T47D human breast cancer cells. *Cancer Res* **48**: 4340–4347

Resnicoff M, Abraham D, Yutanawiboonchai W, Rotman HL, Kajstura J, Rubin R, Zoltick P and Baserga R (1995) The insulin-like growth factor I receptor protects tumor cells from apoptosis in vivo. *Cancer Res* **55**: 2463–2469

Rohlik Q, Adams D, Kull FC Jr and Jacobs S (1987) An antibody to the receptor for insulin-like growth factor I inhibits the growth of MCF-7 cells in tissue culture. *Biochem Biophys Res Commun* **149**: 276–281

Steele-Perkins G, Turner J, Edman JC, Hari J, Pierce SB, Stover C, Rutter WJ and Roth RA (1988) Expression and characterization of a functional human insulin-like growth factor I receptor. *J Biol Chem* **263**: 11486–11492

Stewart AJ, Johnson MD, May FE and Westley B (1990) Role of insulin-like growth factors and the type I insulin-like growth factor receptor in the oestrogen-stimulated proliferation of human breast cancer cells. *J Biol Chem* **265**: 21172–21178

Stewart AJ, Westley B and May F (1992) Modulation of the proliferative response of breast cancer cells to growth factors by oestrogen. *Br J Cancer* **66**: 640–648

Thorsen T, Lahooti H, Rasmussen M and Aakvaag A (1992) Oestradiol treatment increases the sensitivity of MCF-7 cells for the growth stimulatory effect of IGF-1. *J Steroid Biochem Mol Biol* **41**: 537–540

Toi M, Harris A and Bicknell R (1993) cDNA transfection followed by the isolation of a MCF-7 breast cell line resistant to tamoxifen in vitro and in vivo. *Br J Cancer* **68**: 1088–1096

Ullrich A, Gray A, Tam A, Yang-Feng T, Tsubokawa M, Collins C, Henzel W, Le Bon T, Kathuria S, Chen E et al (1986) Insulin-like growth factor I receptor primary structure: comparison with insulin receptor suggests structural determinants that define functional specificity. *EMBO J* **5**: 2503–2512

van den Berg HW, Claffie D, Boylan M, McKillen J, Lynch M and McKibben B (1996) Expression of receptors for epidermal growth factor and insulin-like growth factor I by ZR-75-1 human breast cancer cell variants is inversely related – the effect of steroid hormones on insulin-like growth factor I receptor expression. *Br J Cancer* **73**: 477–481

Werner H, Woloschak M, Adamo M, Shen-Orr Z, Roberts CT Jr and LeRoith D (1989) Developmental regulation of the rat insulin-like growth factor I receptor gene. *Proc Natl Acad Sci USA* **86**: 7451–7455

Westley BR and May F (1994) Role of insulin-like growth factors in steroid modulated proliferation (Review). *J Steroid Biochem Mol Biol* **51**: 1–9

Wiseman LR, Johnson MD, Wakeling AE, Lykkesfeldt AE, May FE and Westley BR (1993) Type I IGF receptor and acquired tamoxifen resistance in oestrogen-responsive human breast cancer cells. *Eur J Cancer* **29A**: 2256–2264

Yee D, Cullen KJ, Paik S, Perdue JF, Hampton B, Schwartz A, Lippman ME and Rosen N (1988) Insulin-like growth factor II mRNA expression in human breast cancer. *Cancer Res* **48**: 6691–6696

 *© Cancer Research Campaign 1999*
